# The yin and yang functions of extracellular ATP and adenosine in tumor immunity

**DOI:** 10.1186/s12935-020-01195-x

**Published:** 2020-04-07

**Authors:** Li-li Feng, Yi-qing Cai, Ming-chen Zhu, Li-jie Xing, Xin Wang

**Affiliations:** 1 Department of Hematology, Shandong Provincial Hospital Affiliated to Shandong University, Shandong First Medical University, Jinan, 250021 Shandong China; 2 School of Medicine, Shandong University, Jinan, 250012 Shandong China; 3 Shandong Provincial Engineering Research Center of Lymphoma, Jinan, 250021 Shandong China; 4 National clinical research center for hematologic diseases, Jinan, 250021 Shandong China; 5 Department of Clinical Laboratory, Nanjing Medical University Cancer Hospital & Jiangsu Cancer Hospital, Nanjing, 210009 Jiangsu China

**Keywords:** Extracellular adenosine triphosphate, Adenosine, CD39, CD73, Tumor immunity

## Abstract

Extracellular adenosine triphosphate (eATP) and its main metabolite adenosine (ADO) constitute an intrinsic part of immunological network in tumor immunity. The concentrations of eATP and ADO in tumor microenvironment (TME) are controlled by ectonucleotidases, such as CD39 and CD73, the major ecto-enzymes expressed on immune cells, endothelial cells and cancer cells. Once accumulated in TME, eATP boosts antitumor immune responses, while ADO attenuates immunity against tumors. eATP and ADO, like yin and yang, represent two opposite aspects from immune-activating to immune-suppressive signals. Here we reviewed the functions of eATP and ADO in tumor immunity and attempt to block eATP hydrolysis, ADO formation and their contradictory effects in tumor models, allowing the induction of effective anti-tumor immune responses in TME. These attempts documented that therapeutic approaches targeting eATP/ADO metabolism and function may be effective methods in cancer therapy.

## Background

Adenosine triphosphate, also known as ATP, is actively released to the extracellular environment in response to tissue damage and cellular stress. The concentration of cellular ATP is 3 to 10 mM. However, the concentration of extracellular ATP (eATP) is only about 10 nM [1]. The concentration gradient is maintained by ecto-nucleotidases, such as CD39 and CD73, which hydrolyze released ATP rapidly to adenosine diphosphate (ADP), adenosine monophosphate (AMP), and then adenosine (ADO) [2]. CD39/ecto-nucleoside triphosphate diphosphohydrolase-1 (ENTPDase1) is the dominant ecto-nucleotidase broadly expressed on immune cells, endothelial cells (ECs) and tumor cells, which drives the sequential hydrolysis of ATP and ADP to AMP [3]. The formation of AMP to ADO is accomplished primarily through ecto-5-nucleotidase (CD73), a glycosyl phosphatidylinositol-linked membrane protein, also expressed on various immune cells, ECs and tumor cells [4].

Hypoxia, acute and chronic inflammation, platelet aggregation and anticancer therapies induce tumor cells death and metabolic changes, leading to the increased concentrations of eATP and ADO in tumor microenvironment (TME) [5]. The accumulation of extracellular nucleotides and nucleosides is negligible in healthy tissues, except in highly secluded compartments, such as synaptic clefts and the interstitium of exercising muscle [6, 7]. However, the increase of eATP and ADO comprises important components in TME, which is a complex system consisting of host derived microvasculature, stroma, endothelial cells, pericytes, fibroblasts, smooth muscle cells and immune cells, characterized further by hypoxia, acidosis and high interstitial fluid pressure [8]. The components in TME communicate with each other, and also with cancer cells, regulating cellular processes which can inhibit or promote tumor growth [9]. They participate in tumor genesis and development through various mechanisms. In TME, eATP boosts antitumor immune responses while ADO attenuates immune response against tumors [10, 11]. eATP and ADO, like yin and yang, represent two opposite aspects from immune-activating to immune-suppressive signals in tumor immunity.

CD39, CD73, ATP and ADO have been proposed as therapeutic targets in oncology. Therapeutic strategies have been developed to modulate eATP/ADO metabolism and related anti-cancer immune responses. In this review, we focused on the effects and related mechanisms of eATP and ADO in tumor immunity, and then discussed the possibility of targeting purnergic signaling in cancer therapy.

### eATP and ATP receptors in TME

Increased eATP in TME exhibits multiple functions in combination with ATP receptors. There are two P2 receptor families: P2X receptors (P2XR, P2X1-7) which are ATP-gated ion channels and P2Y receptors which are G protein-coupled receptors (P2YR, P2Y1, 2, 4, 6, 11–14) [12]. Different P2 receptors have different affinity/specificity according to expressed cells, whereby modulating different cellular functions [13].

P2X1, 4, 5 receptors were found to express on multiple tumor cells and host cells, especially in hematological malignancies, including acute lymphocytic leukemia, acute myeloid leukemia and multiple myeloma [14, 15]. Their activation mainly associated with the intracellular Ca^2+^ and Na^+^ increase. In hematology malignancies and solid tumors, the activation of P2X7R not only increased intracellular Ca^2+^ and Na^+^ but also decreased intracellular K^+^ [14]. The roles of ATP were depending upon the concentration of eATP in TME. Low eATP levels were anticipated to promote cancer proliferation and immunosuppression, while high levels would promote antitumour immunity [14].

Different P2YR subtypes are activated by different nucleotides. ATP is the preferred ligand only for P2Y11R. For other P2YRs, pyrimidine nucleotides and ADP are more potent agonists. The expression of P2Y11R was found on Huh7 and HepG2 hepatocellular carcinoma cells, hematopoietic stem cells (HSCs) and dendritic cells (DCs) [14]. P2YRs are G protein-coupled and usually activate phospholipase C-IP3 pathway which modulates endoplasmic reticulum Ca^2+^ release. Three Gi/o-coupled subtypes (P2Y12, P2Y13 and P2Y14) mainly inhibit adenylyl cyclase to regulate cyclic AMP (cAMP)/protein kinase A (PKA) pathway [16, 17].

### eATP in tumor immunity

**Fig. 1:**
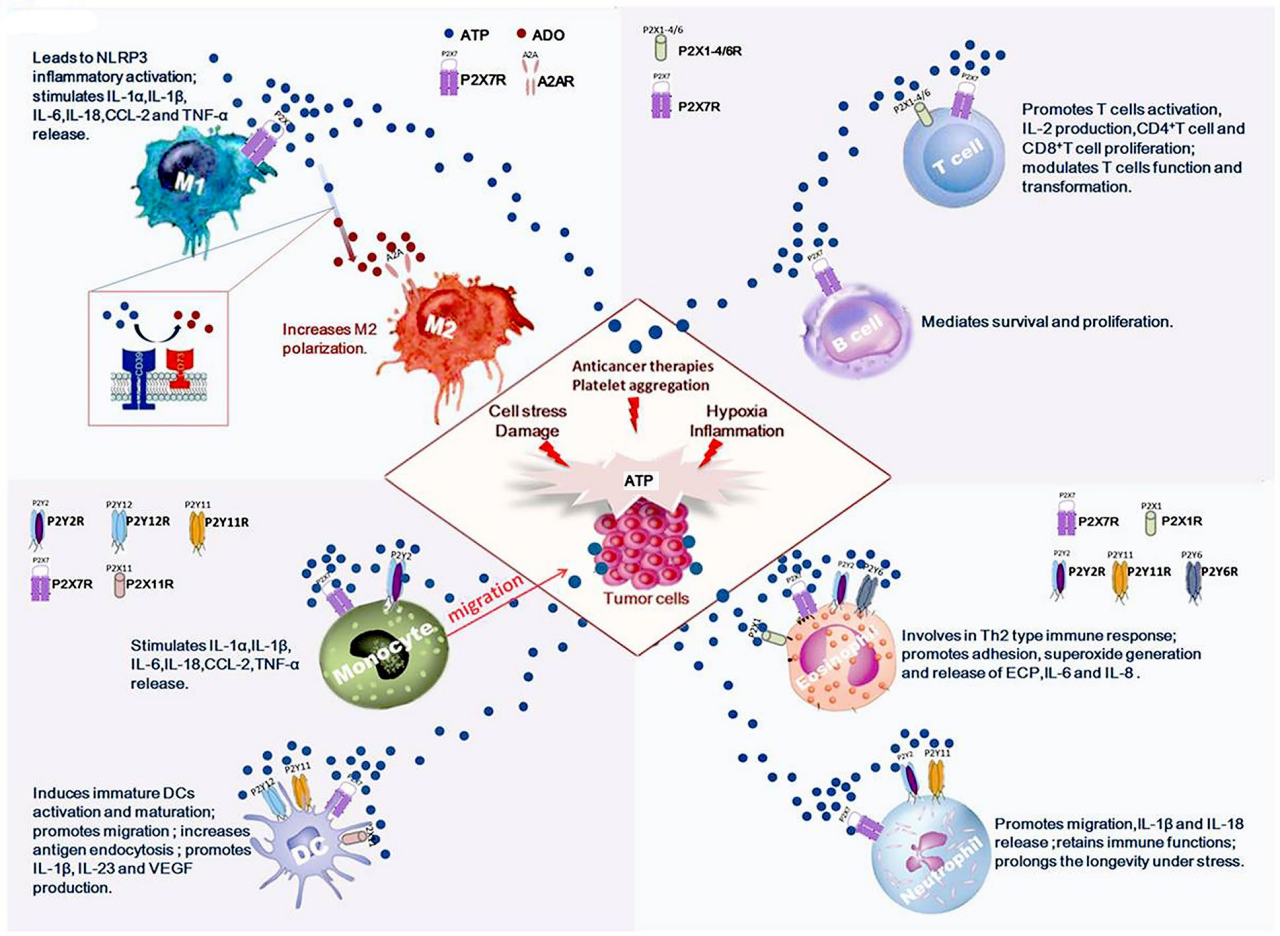
Tissue damage, cellular stress, hypoxia, acute and chronic inflammation, platelet aggregation and anticancer therapies induce tumor cells death and metabolic changes, leading to the increased concentrations of extracellular ATP (eATP) and adenosine (ADO) in tumor microenvironment (TME) (3 to 10 mM). eATP communicates with components of TME to play the immune-activating functions, including monocytes (Mo)/macrophages (Mø), dendritic cells, T lymphocytes, eosinophils, neutrophils and B cells

Contemporary perspectives highlight the roles of TME in tumor growth and therapy. Increasing evidence indicated that targeting TME could complement traditional treatment and improve therapeutic outcomes for malignancies. As a major component, eATP in TME is at a high concentration [18, 19]. The activation of P2X7R mediates high concentrated ATP-induced tumor cells death directly [20]. Besides the direct inhibition of tumor growth, eATP communicates with other immune cells in TME to regulate tumor growth (Fig. 1).

#### eATP and monocytes (Mo)/macrophages (Mø)

Tumor-associated macrophages (TAMs) are key members in TME. Macrophages (Mø) play different roles in tumor growth depending on their polarization to classically-activated macrophages (M1s, release tumor necrosis factor-alpha (TNF-α)) or alternatively-activated macrophages (M2s, release IL-10) [21]. M1s conduct the pro-inflammation and anti-tumor effects, while M2s mainly exhibit anti-inflammation and tumor promoting effects. M2s play important roles in cancer cell survival, proliferation, stemness and invasiveness, along with angiogenesis and immunosuppression [22]. M1 cells have a low expression of ecto-nucleotidases and rate of ATP hydrolysis as compared to M2 cells [23]. The effects of eATP on Mo/Mø also depend on the levels of eATP and receptor subtypes. eATP at a high concentration functions as a danger signal activating P2X7R and leading to the activation of the NLRP3 proinflammatory inflammasome pathway in M1s [24]. Low levels of eATP and other nucleotides, such as ADP and UTP, activate the G-protein-coupled P2YRs to mediate chemotaxis of myeloid cells to damaged tissues [25]. eATP at micromolar concentration also fuctions as a chemoattractant for Mo migration to tumor [26]. In addition, eATP has a considerable impact on cytokine production from Mo/Mø. eATP at millimolar concentration stimulates production of IL-1α, IL-1β, IL-6, IL-18, chemokine (C–C motif) ligand (CCL)-2 and TNF-α [27]  via P2X7 receptor expressed on Mo/Mø [28, 29]. Recently, accumulated evidences supported the roles of multiple P2XR and P2YR subtypes in Mo/Mø, including P2X1R, P2X4R, P2X5R, P2X7R, P2Y2R, P2Y4R, P2Y6R, P2Y11R, P2Y13R and P2Y14R, which potentially conduct cytokine release and phagocytosis respectively [30]. Macrophage differentiation in the presence of ADO results in an M2-like phenotype [24]. The effects of extracellular nucleotides on M2 cells are mainly mediated by ADO (described below).

#### eATP and DCs

DCs are the most important antigen presenting cells (APCs) playing a pivotal role in tumor immunity. eATP not only affects DCs maturation and migration, but also contributes to the antigen endocytosis, cytokine production and ectonucleotidase expression. eATP induces up-regulation of activation and maturation markers (i.e. CD80, CD83, CD86, CD54, human leukocyte antigen DR (HLA-DR) and major histocompatibility complex (MHC)-II) in human peripheral blood mononuclear cell (PBMC)-derived immature DCs, possibly by stimulation of P2Y11R and P2X7R [31]. Maturating DCs then migrate from peripheral tissues to T cells areas to deliver antigens and immunomodulatory messages [32]. Activation of P2Y12R increases antigen endocytosis of DCs with subsequent T cells activation [33]. eATP also has effects on cytokine production from DCs. It has been reported that eATP triggers NOD-like receptor family NLRP3-dependent caspase-1 activation complex (“inflammasome”), allowing the secretion of IL-1β through activation of P2X7R on DCs [34]. eATP is also found to promote IL-23 and vascular endothelial growth factor (VEGF) production from DCs in vitro, which is potentially mediated by P2Y11R [31, 35]. Higher IL-23 is essential for Th17 polarization and magnifying Th17-promoting activity of DCs [31]. However, when there is low level of eATP (uM), TNF-α, IL-12 and IL-1 from DCs are completely abrogated [36]. It was recently reported that P2X7R was able to influence the expression of CD39 and CD73 on DCs. P2X7 pharmacological blockade leads to a reduction of tumor volume and an increase of extracellular ATP with conventional DCs down-modulate CD39 and CD73 [37]. In acute myeloid leukemia patients and mice, it was found that ATP release from chemotherapy-treated dying cells increased number of IDO1^+^CD39^+^ DCs [38].

#### eATP and T cells

Recent studies have shown that eATP regulates T cell activation, cytokine production and lymphocyte metabolism [39, 40]. P2X receptor subtypes are found to involve in T-cell activation which requires a sustained elevation of intracellular Ca^2+^ levels. All P2XRs, with the exception of P2X5, can facilitate entry of Ca^2+^ in response to stimulation by eATP, suggesting that P2X receptors regulate T-cell activation by increasing intracellular Ca^2+^ [41]. P2XRs activation by ATP also protracts the T cell receptor (TCR)-initiated activity of MAPKs and secretion of IL-2 [42]. In contrary, removal of eATP or inhibition, mutation or silencing of P2X1, P2X4 or P2X7 receptors inhibits Ca^2+^ entry, nuclear factors of activated T cells (NFAT) activation and T-cell activation [41, 43–45]. For T cells, P2X7R is found to distribute uniformly across the cell surface and can be activated by high concentration of eATP, occurring in damaged tissue and under cellular stress. This feature of P2X7R facilitates T cells to distinguish damaged tissues, especially in TME [46]. P2X7R also plays important roles in eATP (100–300 μM) stimulated mitogen-induced CD4^+^ and CD8^+^ T cells proliferation. eATP also acts as a potent chemoattractant for T cells through P2YRs signaling [36]. Extracellular ATP produced by damaged tissue or exported by activated cells, is found not only triggers immune activation but plays an additional critical role by promoting metabolic fitness and survival of the most durable and functionally relevant memory CD8^+^ T cell populations through the activation of P2X7R [40].

Extracellular ATP and its receptor P2X7 also exert a pivotal influence on cancer growth through modulating lymphocytes infiltration in TME, including CD4^+^, CD8^+^ lymphocytes and regulatory T cells (Tregs). Elena De Marchi et al. [37] reported that tumors growing in P2X7 null mice showed a decrease in CD8^+^ cells and an increased number of Tregs, overexpressing the fitness markers OX40, PD-1, and CD73. However, systemic administration of the P2X7 blocker A740003 in wild-type mice showed increased CD4^+^ effector cells and decreased their expression of CD39 and CD73. These data illustrated that P2X7 receptor was a key determinant of TME composition.

In addition, eATP is a modulator for T cells function transformation. Antagonists of P2XRs can promote the cell-autonomous conversion of naive CD4^+^ T cells to Tregs after TCR stimulation [41]. The activation of P2X7R not only reduces Treg cells numbers, but also inhibits Treg cells generation, suppressive function and stability [38]. eATP released by commensal bacteria was also found to drive the differentiation of intestinal CD4^+^ T cells to Th17 cells [1]. Another study confirmed that the activation of P2X7R on follicular helper T (Tfh) cells weakened germinal center reactions and immune globulin (Ig) A secretion, and resulted in increased serum IgM [46].

#### eATP and eosinophils, neutrophils and B cells

The participation of eosinophils in innate Th2-type immune responses is mediated by eATP [47]. eATP also promotes eosinophils functions through P2Y2R, including adhesion and superoxide generation [48]. Human eosinophils release eosinophil cationic protein (ECP), IL-6 and IL-8 by P2Y2 receptor stimulation, while P2X1, P2X7, and P2Y6 receptors might also be implicated in the release of IL-8 [47, 49]. In hypoxia, neutrophils contribute to production of eATP. In return, the activation of P2Y2R by eATP promotes neutrophils migration to chemoattractants [50]. Neutrophils also express functional P2X7R, involving in the secretion of IL-1β and IL-18 [51]. In addition, activation of P2Y11R helps neutrophils to retain the immune functions and prolong the longevity under stress [30]. For B cells, the activation of P2X7R mainly mediates B-cell survival and proliferation [52].

### ADO and ADO receptors in TME

Similar to eATP, the concentration of ADO is at a low level in extracellular media (lower than 1 μM). However, its concentration notably increases under many metabolically stressful conditions, especially in TME (over 100 μM) [19]. The bulk of extracellular ADO is generated from eATP thanks to sequential hydrolysis of CD39 and CD73 [17]. ADO binds to its receptors (A1, A2A, A2B, and A3) presented on immune cells and tumor cells, regulating tumor progression and multiple immune responses, including tumor immunity [53]. A2A and A2B receptors (A2AR and A2BR) are Gs-coupled receptors that increase intracellular cAMP and PKA levels, playing dominant roles in ADO-induced immunosuppression in a cAMP-dependent manner [54]. A1 and A3 receptors (A1R and A3R) are Gi/o-coupled receptors that decrease intracellular cAMP, thereby favoring cell activation but also inducing activation of phosphatidylinositol 3-kinase (PI3K), extracellular-signal-regulated kinase (ERK)1/2 and protein kinase C (PKC) [55, 56]. Receptors with high-affinity (A1R, A2AR and A3R) are involved when ADO is at low concentrations, whereas at high concentrations, the low-affinity A2BR is involved, like those observed in TME [57].

ADO affects tumor progression directly through binding on its specific receptors expressed on cancer cells. Extracellular ADO in TME chronically activates A2BR expressed on cancer cells to suppress Ras-related protein (Rap)-1B prenylation and signaling, resulting in cell–cell contact reduction and cell scattering promotion. The major effect of A3R activation is to promote angiogenesis [58].

### Extracellular ADO in tumor immunity

**Fig. 2:**
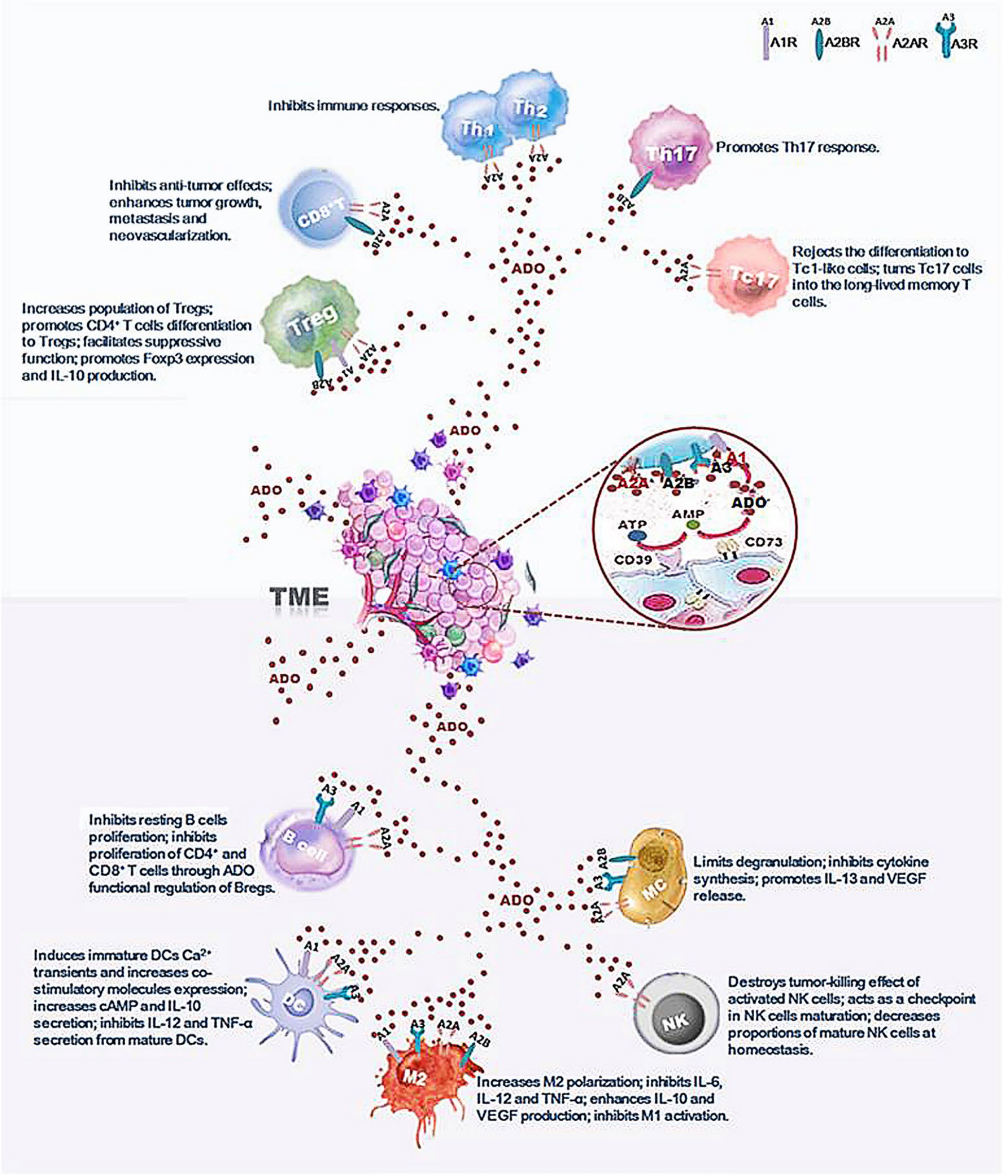
Extracellular adenosine (ADO) at a high concentration (over 100 μM) conducted immune-suppressive functions through activation of different ADO receptors on immune cells in tumor microenvironment (TME). For T cells in TME, ADO not only decreases anti-tumor function of CD8+ T cells, Th1 cells and Th2 cells but also enhances the function of regulatory T cells (Treg), Th17 cells and Tc17 cells. ADO also affects B cells, dendritic cells (DCs), mast cells (MCs), natural killer (NK) cells and macrophages (Mø) functions in anti-tumor immunity

Extracellular ADO in TME is also an important factor controlling antitumor immunity, conditioning both innate and adaptive immune responses (Fig. 2).

#### ADO and T cells

ADO is emerging as an important negative regulator of T-cell function for inhibition of mobility, migration and adhesion [59–61]. The inhibition role of ADO in anti-tumor T cells is mediated by A2AR and A2BR. Pharmacological blockade of A2AR not only enhances CD8^+^ T cells anti-tumor response but also reduces the population of Tregs [62]. A2AR antagonist or silence by siRNA could improve the inhibition of tumor growth, destruction of metastases and prevention of neovascularization by anti-tumor T cells [63]. For Treg cells, ADO not only promotes CD4^+^ T cells differentiation to Tregs [17], but also facilitates Treg cells suppressive function through A2AR activation [64]. Antagonists of A1R and A2BR also suppress the expression of Foxp3 and the production of IL-10 from Tregs [65]. Moreover, human Tregs over-express CD39 and CD73, which are beneficial to ADO production. ADO then binds to A2AR on effector T cells and suppresses their functions [66]. Meanwhile, Treg cells express low levels of adenosine deaminase (ADA), the enzyme responsible for ADO breakdown, and CD26, a surface-bound glycoprotein associated to ADA, which is helpful to maintain the high concentration of ADO in TME [67]. A2AR activation on T cells under hypoxia could also suppress the development of Th1 and Th2 immune responses, both in vitro and in vivo [68]. A2BR is found to play important roles in the effects of ADO on Th17 cells. A2BR activation promotes Th17 response through DCs activation and differentiation from bone marrow cells to a CD11c^+^Gr-1^+^ DC subset [69, 70]. Another subset of T cells, named Type 1 cytotoxic CD8^+^ T (Tc1) cells, is latterly recognized as IL-17-producing CD8+ T (Tc17) cells. It is demonstrated that Tc17 cells stably sustain the stem-cell-like program and reject the differentiation to Tc1-like cells with the help of ADO, consequently activating A2AR, which turns Tc17 cells to long-lived memory T cells to allow the immunosuppressive properties in tumor progression [71].

#### ADO and B cells

Abundant expression of CD39 and CD73 on human B cells facilitates an efficient accumulation of ATP-generated ADO, thus contributing to T cell-B cell interactions [72]. B cells have been found to express A1R, A2AR and A3R, but not A2BR. Agonists and antagonists studies were conducted to determine which ADO receptor was involved in B cells suppressive signals delivery. A selective A3R antagonist was found to stimulate proliferation of resting B-cells while a A3R agonist inhibited their proliferation, indicating that ADO produced by resting B cells exerted autocrine action [73]. Activated human B cells could also down-regulate proliferation of autologous CD4^+^ and CD8^+^ T cells through ADO [73]. Recently, a new paradigm of regulatory B cells (Bregs) was introduced [74] and they were thought to be able to control CD4^+^ T-cell responses [75]. Functional regulation of Bregs is manipulated by different ADO receptors respectively. A1R and A2AR contribute to the proliferation and functions of CD39^high^ B cells while A2BR activation is critically required for IL-10 production [76].

#### ADO and DCs

ADO, an important modulator of DCs functions, has significant effects on DCs activation, maturation, as well as cytokine production. A1R, A2AR and A3R are expressed by human monocyte-derived DCs. For immature DCs, ADO not only induces Ca^2+^ transients resulting in actin polymerization and chemotaxis, but also increases co-stimulatory molecules expression [30]. For mature DCs, ADO increases cAMP and IL-10 secretion while inhibits IL-12 and TNF-α production [77]. ADO is also found to activate human monocyte-derived DCs through A2AR [78] and murine bone marrow-derived dendritic cells (BMDCs) through A2BR [79]. However, unlike normal myeloid DCs, ADO-differentiated DCs have impaired allostimulatory activity and express high levels of angiogenic, pro-inflammatory, immune suppressor and tolerogenic factors [80]. ADO-conditioned DCs also have potential to regulate polarization of some T-cell subtypes. In vitro, ADO-conditioned DCs reduce the capacity of Th1 polarization from CD4^+^ T cells in a cAMP-dependent manner [81]. DCs from A2BR agonist-treated mice showed a significantly increased ability to activate γδ T cells and Th17 autoreactive T cells [69]. Moreover, ADO deaminase knockout animal models showed that elevated levels of extracellular ADO were highly associated with DCs with a pro-angiogenic phenotype [82]. Therefore, a defective function of DCs under the activation of ADO-A2AR/A2BR axis in TME allows tumor to escape immune surveillance.

#### ADO and mast cells (MCs)

The amount of tumor-infiltrating MCs has a close relationship with tumor aggressiveness and dissemination [83]. A2AR, A2BR, and A3R are found to be expressed on MCs. A2BR activation limits cells degranulation [84]. The combination of A2AR and A2BR activation is required for the inhibition of cytokine synthesis both in vitro and in vivo [85, 86]. MCs isolated from wild type (WT), not A2BR knocked out (KO) mice, release IL-13 and VEGF in response to extracellular ADO, suggesting the role of A2BR in angiogenesis [87]. However, A3R activation has been proven to mediate MCs activation through ERK1/2 phosphorylation in TME [88].

#### ADO and natural killer (NK) cells

For NK cells, which are a type of innate immune cells, increased levels of adenosine in tumor environment inhibit the lytic activity of NK cells via binding to A2A receptors [89]. A2AR deletion increases proportions of mature NK cells at homeostasis, following reconstitution in TME. Targeting A2AR specifically on NK cells delays tumor genesis, suggesting A2AR-mediated signaling as an intrinsic negative regulator for NK cells maturation and antitumor immune responses [90].

#### ADO and Mø

Increased ADO concentration could also induce Mø recruitment in TME. Mø plays various roles in an ADO-dependent manner [91]. A1R, A2AR, A2BR and A3R are all expressed on Mø cells. But the effects of ADO on Mø cells are predominantly mediated by A2AR. As described before, Mø cells played opposite roles depending on their polarization to M1s or M2s. A2AR activation leads to the increase of cAMP and the activation of CCAAT-enhancer-binding protein-β, thereby increase M2 macrophage polarization [92]. ADO also inhibits classical Mø activation [93]. For cytokine production, ADO not only inhibits TNF-α, IL-6, and IL-12 release but also enhances IL-10 and VEGF production from Mø [94, 95].

### Targeting purinergic signaling as potential target in cancer therapy

Given the yin and yang function of eATP and ADO in tumor immunity, targeting their signaling pathways and their metabolism has attracted more and more attention in tumor therapy.

#### P2 receptors as tumor therapeutic targets

Among P2Rs, P2X7R is the most potential candidate in anticancer therapy [96]. P2X7R was reported to be expressed in multiple malignant tumors, including neuroblastoma [97], melanoma [98], prostate cancer [99], lung cancer [100] and breast cancer [101]. P2X7R antagonists could inhibit tumor growth and migration [96]. With pretreatment of P2X7R antagonists (including A-740003, A438079 and KN62), ATP-evoked tumor growth (ATP < 1.0 mM) was inhibited via PI3K/AKT and AMP-activated protein kinase (AMPK)/mTOR pathways [97, 102, 103]. Another P2X7R antagonist, named AZ10606120, was found to execute growth-inhibitory effect in neuroblastoma through PI3K/GSK3β/hypoxia-inducible factor 1-alpha (HIF-1α) pathway downregulation [104]. Moreover, P2X7R antagonists could affect inflammatory cytokines secretion. A740003 not only inhibited IL-10, IL-6 and monocyte chemoattractant protein-1 (MCP-1) release from macrophage [77], but also reduced cancer antigen presentation and cancer cell proliferation [105]. By down-regulated CXCL1, MCP-1 and VEGF secretion, P2X7R antagonist (such as BBG and AZ10606120) contributed to inhibition of endothelial progenitor cells (EPCs) as well as vascular density and tumor neovascularization [106, 107]. Another form of P2X7R, which is not able to form a functional pore and termed as non-pore functional P2X7R (nfP2X7R), has been described recently [101]. Researchers found that antibodies against amino acid sequence specifically for nfP2X7R also had a key role in cancer growth [108], which documented that antagonists specifically for nfP2X7R may be potential effective therapeutic agents in cancer therapy.

P2Y12R is another important member in P2R family. Under ATP and ADP stimulation, P2Y1R initiates platelet activation followed by the ADP-P2Y12R-mediated amplification [109]. P2Y12R represents a potential target in cancer therapy due to its involvement in platelet-cancer cell crosstalk. Thus, P2Y12R antagonists, including clopidogrel, ticagrelor and prasugrel, might represent potential anti-cancer agents [110].

#### ADO receptors as tumor therapeutic targets

Strategies targeting ADO receptors appear to be attracting approaches to block ADO immunosuppressive effects and boost anti-tumor immunity. In a sarcoma model and the poorly immunogenic LL-LCMV tumor model, pharmacologic blockade of A2AR enhanced T cell mediated tumor regression [63]. The ability of A2AR blockade to suppress metastatic has also been validated [111]. A2AR blockade and programmed death-1 (PD-1)/cytotoxic T-lymphocyte–associated antigen 4 (CTLA-4) monoclonal antibody (mAb) combined therapy also illustrated impressive effects in a variety of syngeneic tumor models [112]. Most recently, it was reported that A2AR blockade had high potential to enhance chimeric antigen receptor (CAR)-T cells efficacy in some cancers [113]. There are some agents targeting the A2AR for cancer immunotherapy in Phase 1 trials, including CPI-444 (Corvus), PBF-509 (Novartis/Pablobiofarma), MK-3814 (Merck), AZD4635 (AstraZeneca/Heptares). Each of these trials includes cohorts receiving monotherapy as well as A2AR blockade in combination with blockade of the PD-1/PD-L1 pathway [114]. A2BR is a low-affinity receptor, but its expression is highly transcriptionally regulated by HIF-1α in TME. When tumor A2BR was blocked, the decreased metastasis was observed in multiple tumors [80, 115]. A2BR inhibitor also showed significant anti-tumor effects when combined with anti-PD-1 and anti-CTLA-4 mAbs [115].

#### CD39 and CD73 as tumor therapeutic targets

Considering critical roles of CD39/CD73 in ATP/ADO metabolism, attempts to block CD39/CD73 activity have been tried in tumor therapy. When compared to CD39 over-expressing mice, colorectal cancer cells and melanoma cells in CD39 deficient models showed significantly slower hepatic and pulmonary metastases [116]. Inhibition of CD39 activity by polyoxometalate1 (POM-1) abrogated production of IL-10 from TAMs, functionally diminished immunosuppressive functions [117]. Even for sarcoma, which lacked effective therapeutic intervention, CD39 inhibitory mAb was proven to have prolonged survival in a lethal metastatic patient-derived sarcoma model [118]. Some pharmaceutical companies are currently developing anti-human CD39 mAb, such as Innate Pharma in France. CD73 blockade was also found to have favorable antitumor effects via modulating immune response in TME. CD73-deficient mice were demonstrated to sustain activated antitumor immunity, influencing neovascularization, tumor growth and metastasis [119, 120]. CD73 inactivation was proved to inhibit progression of breast cancer and head and neck squamous cell carcinoma [121, 122]. Likewise, anti-CD73 blocking antibody was also documented to impact spontaneous lung metastasis and decrease circulation capability of cancer cells [4]. Anti-CD39 and anti-CD73 monoclonal antibodies and their combination with immune checkpoint inhibitors and chemotherapies in cancer also showed promoted antitumor immunity [123]. CD73 inhibitors alone and in combination with other drugs in cancer patients are currently under clinical trials (such as NCT03454451, NCT02503774 and NCT03381274 registered at clinicaltrials.gov).

## Conclusions and future perspectives

Purinergic signaling has emerged as a novel mechanism to modulate anticancer immunity. The accumulations of eATP and ADO in TME play yin and yang functions in tumor immunity through their specific receptors. The metabolism from eATP to ADO is accomplished mainly through CD39 and CD73, which expressed on immune cells, endothelial cells as well as cancer cells in TME. Drugs and biological approaches blocking eATP hydrolysis, ADO formation and their effects have the potential to overcome tumor immunosuppression, allowing the induction of effective anti-tumor immune responses. This strategy could also be a new option to improve the efficacy of cytotoxic agents and checkpoint blockade inhibitors. However, given the multifaceted effects of eATP and ADO in TME, where tumor cells, host immune cells and stromal cells communicate with each other through complicated mechanisms, how to select the most suitable therapy target is still a challenge to be overcome.

## Data Availability

No datasets were generated or analysed

## Abbreviations

ADO: Adenosine
ADP: Adenosine diphosphate
AMP: Adenosine monophosphate
AMPK: AMP-activated protein kinase
APCs: Antigen presenting cells
ATP: Adenosine triphosphate
BMDCs: Bone marrow-derived dendritic cells
Bregs: Regulatory B cells
cAMP: Cyclic adenosine monophosphate
CAR: Chimeric antigen receptor
CCL: Chemokine (C–C motif) ligand
CTLA-4: Cytotoxic T-lymphocyte–associated antigen 4
DCs: Dendritic cells
eATP: Extracellular adenosine triphosphate
ECs: Endothelial cells
EPCs: Endothelial progenitor cells
ERK: Extracellular-signal-regulated kinase
HIF-1α: Hypoxia-inducible factor 1-alpha
HLA-DR: Human leukocyte antigen DR
HSC: Hematopoietic stem cells
Ig: Immune globulin
IL: Interleukin
KO: Knocked out
mAb: Monoclonal antibody
MCP-1: Monocyte chemoattractant protein-1
MCs: Mast cells
MHC: Major histocompatibility complex
Mo: Monocytes
Mø: Macrophages
NFAT: Nuclear factors of activated T cells
nfP2X7R: Non-pore functional P2X7R
NK: Natural killer
PBMC: Peripheral blood mononuclear cell
PD-1: Programmed death-1
PI3K: Phosphatidylinositol 3-kinase
PKA: Protein kinase A
PKC: Protein kinase C
POM-1: Polyoxometalate1
P2XR: P2X receptors
P2YR: P2Y receptors
Rap: Ras-related protein
TAMs: Tumor-associated macrophages
TCR: T cell receptor
Tfh: Follicular helper T cell
TME: Tumor microenvironment
TNF-α: Tumor necrosis factor-alpha
Tregs: Regulatory T cells
VEGF: Vascular endothelial growth factor
WT: Wild type
